# Volume Stabilization of Steel Slag for Low-Carbon Construction Materials: Mechanisms, Treatment Technologies, and Engineering Application Pathways

**DOI:** 10.3390/ma19194181

**Published:** 2026-09-30

**Authors:** Jiarui Gu, Zinuo Tian, Yujie Shao, Xiaoming Liu, Zengqi Zhang, Yinming Sun

**Affiliations:** 1 State Key Laboratory of Advanced Metallurgy, University of Science and Technology Beijing, Beijing 100083, China; 2 School of Metallurgical and Ecological Engineering, University of Science and Technology Beijing, Beijing 100083, China; gjr18832313078@126.com (J.G.); a1183011763@163.com (Z.T.); syj001114@163.com (Y.S.)

**Keywords:** steel slag, volume stability, modification mechanism, application

## Abstract

**Highlights:**

- Formation and delayed hydration mechanisms of f-CaO and f-MgO in steel slag were summarized.
- Surface, high-temperature, acid, and carbonation stabilization technologies were reviewed.
- Mechanisms, advantages, limitations, and application scenarios of stabilization routes were compared.
- Engineering performance of stabilized steel slag in construction materials was evaluated.
- Future pathways for synergistic stabilization and standardized assessment were proposed.

**Abstract:**

Steel slag (SS), a major industrial solid waste generated during steelmaking, has considerable cementitious potential and high hardness for use in low-carbon construction materials. However, its widespread utilization is limited by poor volume stability arising from the delayed hydration and expansion of free CaO (f-CaO) and free MgO (f-MgO). This review systematically examines the formation mechanisms of these expansive phases and evaluates four stabilization technologies, including surface modification, high-temperature modification, acid treatment, and carbonation, with respect to their reaction mechanisms, technical advantages, limitations, and engineering applicability. The performance of stabilized SS in cementitious matrices and as construction aggregates is also assessed, together with the corresponding application scenarios. Carbonation is a promising route because it can simultaneously reduce reactive expansive phases and mineralize CO_2_. However, its stabilization efficiency depends strongly on slag characteristics, reaction conditions, and achievable carbonation depth. Major barriers to industrial application include reliance on single-modification strategies, limited engineering-scale validation, and the lack of unified standards for evaluating volume stability. Future research should focus on synergistic composite technologies, particularly flue-gas carbonation coupled with microbial mineralization, and standardized multi-indicator evaluation frameworks to promote the large-scale, high-value, and low-carbon utilization of SS.

## 1. Introduction and Review Methodology

### 1.1. Introduction

**Figure 1:**
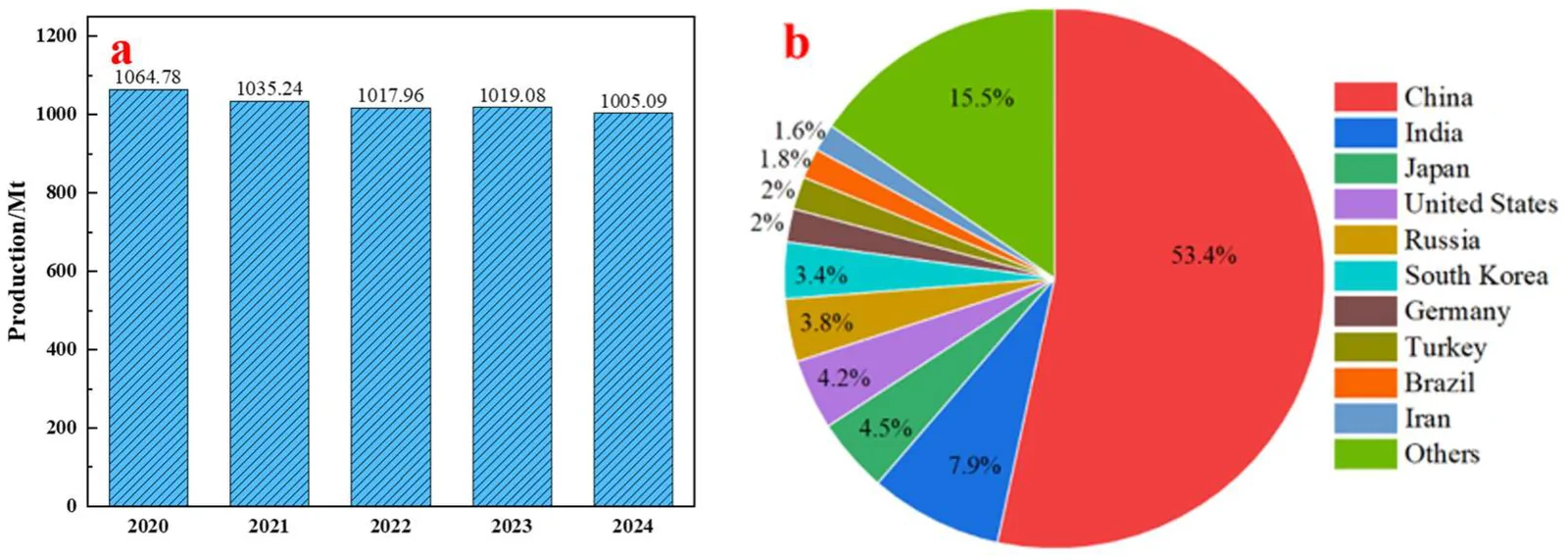
Crude steel production: (**a**) crude steel production in China (2020–2024), (**b**) schematic diagram of crude steel production of major countries worldwide in 2024 [8].

Steel slag (SS), a predominant industrial solid waste generated in steelmaking processes, is closely linked to the output of iron and steel in terms of its production volume [1,2]. Statistically, the annual crude steel output of China has exceeded 1 billion tons in the past five years, accounting for over half of the global crude steel production (Figure 1). The production of one ton of crude steel generates 0.15 to 0.20 tons of SS, while the comprehensive recycling rate of SS in China remains below 40% [3,4,5]. This indicates that a large quantity of SS is not effectively processed. The substantial stockpiling of SS not only wastes land resources and causes severe environmental pollution but also represents a significant waste of potential resources [6,7].

**Figure 2:**
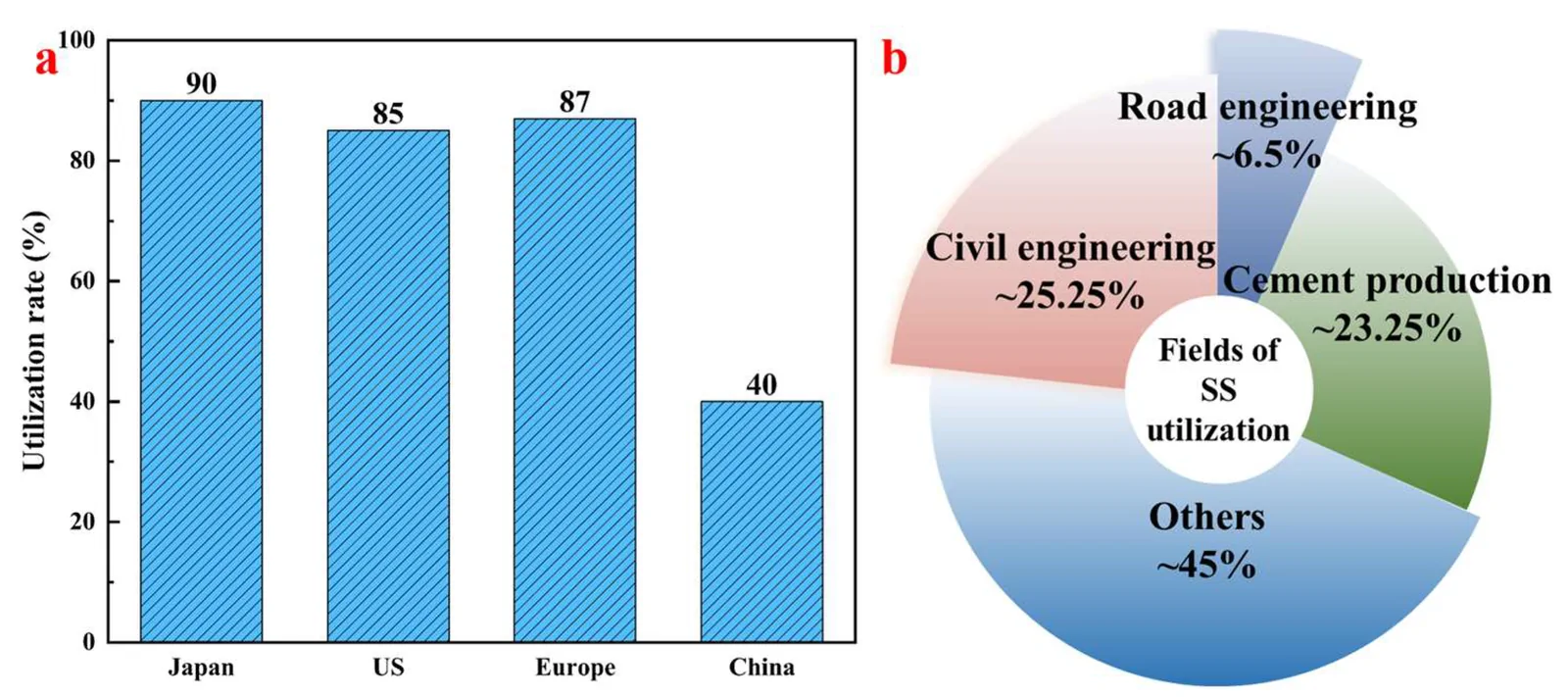
(**a**) The utilization rates of SS in various countries, (**b**) Fields of SS utilization in China [3].

At the beginning of the 20th century, developed countries including the United States, Germany and Japan initially carried out research on the disposal and recycling of SS and formulated comprehensive management regulations for industrial solid wastes [9]. At present, these countries have accumulated abundant practical experience in the comprehensive utilization of SS. In particular, SS has been widely utilized as metallurgical feedstock, road base material, and raw material for cement production, and is already extensively recycled in road construction projects and within steel mills. The utilization rate of SS is over 90% in Japan, approximately 85% in the United States, and 87% in Europe, while that in China is relatively low at approximately 40% (Figure 2) [7,10,11,12]. This is primarily due to the fact that China mainly employs long-process converter steelmaking, resulting in SS with high levels of f-CaO/MgO, significant compositional fluctuations, and an extremely high risk of expansion. In contrast, Europe, the US, and Japan rely heavily on short-process electric arc furnace steelmaking, which produces SS with greater stability. Although domestic iron and steel enterprises and researchers have actively introduced and developed advanced technologies to improve the comprehensive utilization rate of SS, the overall improvement effect is still relatively limited and has not yet reached the ideal level.

Dicalcium silicate (C2S) and tricalcium silicate (C3S) account for more than 50% of the total mass fraction in the mineral composition of SS, and these two minerals exhibit excellent hydraulic cementitious properties [13,14]. In addition, minerals such as silicates, ferroaluminates and aluminates show similar properties. In principle, SS could partially replace cement as a supplementary cementitious material, reducing both the carbon footprint of construction and the demand for natural aggregates. However, restricted by its poor volume stability, SS has not been widely used in building materials such as cement and concrete. The instability arises from f-CaO and f-MgO, which form during high-temperature steelmaking when excess lime and dolomite are added to remove impurities. These oxides develop dense, slow-reacting crystal structures that hydrate over months or years, long after the surrounding matrix has hardened. The hydration of f-CaO to Ca(OH)_2_ produces a volume expansion of approximately 98%; the hydration of f-MgO to Mg(OH)_2_ produces approximately 148% [15,16]. The resulting internal stress cracks the hardened material, creating pathways for further water ingress and accelerating degradation. In addition, the P_2_O_5_ component present in SS inhibits the formation of high-activity C3S mineral phases [17]. Therefore, it is necessary to develop more advanced pretreatment or application methods to address the various issues associated with SS in these fields.

A broad research effort has sought to overcome this barrier through physical, chemical, and composite modification strategies, including high-temperature calcination, autoclave curing, external admixture activation, and mineral phase reconstruction. However, relevant findings remain scattered across the literature. Existing studies typically focus on a single treatment method or a specific application scenario, and no systematic comparison of technical feasibility, economic efficiency, and engineering applicability has been conducted for mainstream treatment strategies. Three key issues still need to be addressed, including revealing the intrinsic stabilization mechanisms for long-term volume stability under practical service conditions, analyzing major technologies from technical, economic and environmental perspectives, and summarizing current limitations to advance combined strategies for industrial transformation of laboratory research.

In light of the above issues, this review systematically examines the mechanisms governing SS volume instability and the technologies developed to mitigate it. The formation and failure pathways of expansive f-CaO and f-MgO are analyzed, and four prevailing modification technologies are compared across their reaction mechanisms, advantages, limitations, and application scenarios. The engineering performance of modified slag in cementitious binders and aggregates is also evaluated. Particular attention is given to carbonation, which offers the distinctive advantage of coupling volume stabilization with CO_2_ sequestration. On the basis of this synthesis, the bottlenecks that currently prevent industrial translation are identified and research directions for synergistic technologies, low-cost carbonation, and standardized evaluation are proposed.

### 1.2. Review Scope and Literature Selection

Relevant literature was retrieved from Web of Science Core Collection/Scopus/Google Scholar, covering publications from 2016 to 2026. The search strategy combined terms related to steel slag and volume stability, including “steel slag”, “basic oxygen furnace slag”, “electric arc furnace slag”, “ladle furnace slag”, “volume stability”, “expansion”, “free CaO”, “free MgO”, “surface modification”, “high-temperature treatment”, “acid treatment”, and “carbonation”. Studies were retained when they (i) addressed the formation or stabilization of expansive phases in steel slag, (ii) reported treatment mechanisms or engineering performance relevant to volume stability, or (iii) provided quantitative indicators such as free-oxide content, expansion, mechanical properties, CO_2_ uptake, or environmental performance. Duplicate studies, publications unrelated to volume stability, and studies lacking sufficient methodological or performance information were excluded. Reference lists of key review articles and primary studies were additionally screened to identify relevant publications. The final synthesis prioritizes peer-reviewed studies with clearly reported treatment conditions and quantitative performance data.

## 2. Production and Properties of Steel Slag

### 2.1. Production of Steel Slag

SS is the main solid by-product of iron and steel smelting. During converter steelmaking, electric arc furnace steelmaking, and molten steel refining, fluxes such as lime and dolomite are added to remove silicon, phosphorus, and sulfur from the hot metal. These fluxes react with impurities to form molten oxide mixtures, which solidify into SS upon cooling. Depending on the furnace type and process stage, SS is classified as basic oxygen furnace slag (BOFS), electric arc furnace slag (EAFS), or ladle furnace slag (LFS). BOFS originates from the rough refining stage in converters, EAFS from electric arc furnace smelting, and LFS from secondary refining of molten steel in ladle furnaces.

### 2.2. Physical and Chemical Properties of Steel Slag

SS is dense, hard, and wear-resistant. Its apparent density ranges from 3.1 to 3.7 g/cm^3^, which is substantially higher than that of natural sand and gravel [18]. The high iron content makes the slag difficult to grind, with a grindability index of approximately 0.7 relative to standard sand. The material exhibits good compressive strength and impact resistance. Its microstructure is predominantly porous, with porosity controlled by the cooling process; its water absorption is generally low.

Chemically, SS is dominated by CaO, FeO and SiO_2_, which together account for approximately 80–85% of its total mass. Minor components include MgO, Al_2_O_3_, MnO, P_2_O_5_, and TiO_2_ [19,20]. Composition varies substantially across furnace types (Table 1). BOFS is calcium-rich (CaO: 38.7–43.2%) with moderate Fe_2_O_3_ (12.8–25.7%). EAFS contains the highest Fe_2_O_3_ (24.2–36.2%) and elevated Al_2_O_3_ (9.6–13.9%). LFS is distinguished by its ultra-high calcium content (CaO: 43.7–65.7%) and very low Fe_2_O_3_ (1.6–17.4%).

In addition to bulk oxide composition, the mineralogical assemblage varies substantially among different types of steel slag and directly controls their hydration activity, expansion behavior, and response to stabilization [21,22,23,24,25,26,27,28,29,30,31,32]. BOFS commonly contains C_2_S, C_3_S, calcium ferrites, the RO solid solution, f-CaO, and f-MgO. EAFS generally contains C_2_S together with Fe-rich phases such as magnetite or RO/spinel phases, while free CaO and MgO contents depend strongly on steelmaking practice. In contrast, LFS is typically Ca- and Al-rich and frequently contains β/γ-C_2_S, mayenite (C_12_A_7_), C_3_A, merwinite, periclase, and, in some cases, f-CaO. These differences explain why stabilization methods cannot be selected independently of slag source and phase assemblage.

## 3. Factors Influencing SS Volume Stability

### 3.1. The Formation of f-CaO and f-MgO

The f-CaO and f-MgO in SS originate from reactions between hot metal and additives during steelmaking. Excess limestone (CaCO_3_) or dolomite (CaCO_3_·MgCO_3_) is added to adjust the molten steel composition and remove sulfur and phosphorus. A portion of these additives is incorporated into the slag, whereas the remainder persists as unreacted free oxides because dissolution reaches saturation before the reactions are complete. Three factors drive f-CaO formation: (1) unreacted lime particles become physically encapsulated within the solidifying slag; (2) free lime forms a solid solution (CaO·FeO·MnO) with iron and manganese, producing dead-burned lime with low reactivity; and (3) C3S decomposes into C2S and f-CaO at high temperatures. Free MgO derives from magnesium-based refractories used for furnace lining protection and repair. These refractory materials react with acidic oxides in the molten steel, and any unreacted MgO that remains becomes f-MgO in the cooled slag.

### 3.2. The Effect of f-CaO on Volume Stability

The hydration of f-CaO is the primary driver of volume expansion for SS. Water reacts slowly with f-CaO to form Ca(OH)_2_, producing a volume expansion of approximately 98% (Equation (1)) [33]. The resulting microcracks provide space for ettringite growth, which promotes further crack propagation and allows additional water penetration, forming a self-reinforcing degradation cycle [34].

(1) f-CaO + H_2_O → Ca(OH)_2_

Because f-CaO forms at temperatures above 1600 °C, its crystal structure is dense and its hydration can continue for months or years. Residual f-CaO thus hydrates long after the matrix has hardened, causing progressive expansion and cracking. The f-CaO in SS exists in three forms: mono-component, binary-component, and ternary-component [35]. Ternary-component f-CaO (CaO·aFeOx·bMnO_γ_) is the dominant source of expansion. Experimental work has shown that increasing Fe and Mn content lowers the hydration activity of ternary f-CaO, with Mn exerting a stronger inhibiting effect than Fe [36].

### 3.3. The Effect of f-MgO on Volume Stability

Free MgO hydrates to form Mg(OH)_2_, with a volume expansion of approximately 148% (Equation (2)) [37]. The f-MgO in SS occurs predominantly as periclase. Similar to f-CaO, it hydrates slowly at ambient temperature and can continue expanding long after the matrix has hardened. The expansion behavior depends on the crystal content, grain size of the periclase, and the curing conditions of the hardened product.

(2) f-MgO + H_2_O → Mg(OH)_2_

## 4. Methods for Improving Volume Stability

The central challenge in SS utilization is the slow hydration of f-CaO and f-MgO, which drives late-stage expansion and internal cracking. Four widely studied strategies for improving volume stability are surface modification, high-temperature treatment, acid treatment, and carbonation. Each operates through a distinct mechanism. Surface modification forms a dense hydrophobic layer on slag particles by physical coating or chemical reaction to block water from contacting internal free oxides. High-temperature modification regulates the cooling of molten slag to alter the crystal morphology and particle size of f-CaO and f-MgO, reduce their abundance, and stabilize silicate phases, thereby optimizing the mineral composition at the source. Acid treatment converts free oxides into stable calcium or magnesium salts through neutralization, and the reaction products can also coat the slag surface and enhance its compactness. Carbonation uses CO_2_ to react with accessible free oxides and reactive mineral phases, forming stable carbonates that reduce the expansion risk while simultaneously mineralizing CO_2_.

In this review, surface stabilization refers to treatments whose dominant action is confined to the exterior of slag particles, such as coating, hydrophobization, and shallow passivation. These treatments mainly restrict water ingress and delay hydration without necessarily consuming internal expansive phases. In contrast, bulk or internal stabilization refers to treatments that alter, consume, or reconstruct reactive phases throughout a substantial fraction of the slag particle or molten slag. High-temperature phase reconstruction is intrinsically a bulk process, whereas acid treatment and carbonation may range from surface-dominated to bulk stabilization depending on reaction depth, particle size, diffusional resistance, and treatment duration.

### 4.1. Surface Modification

**Figure 3:**
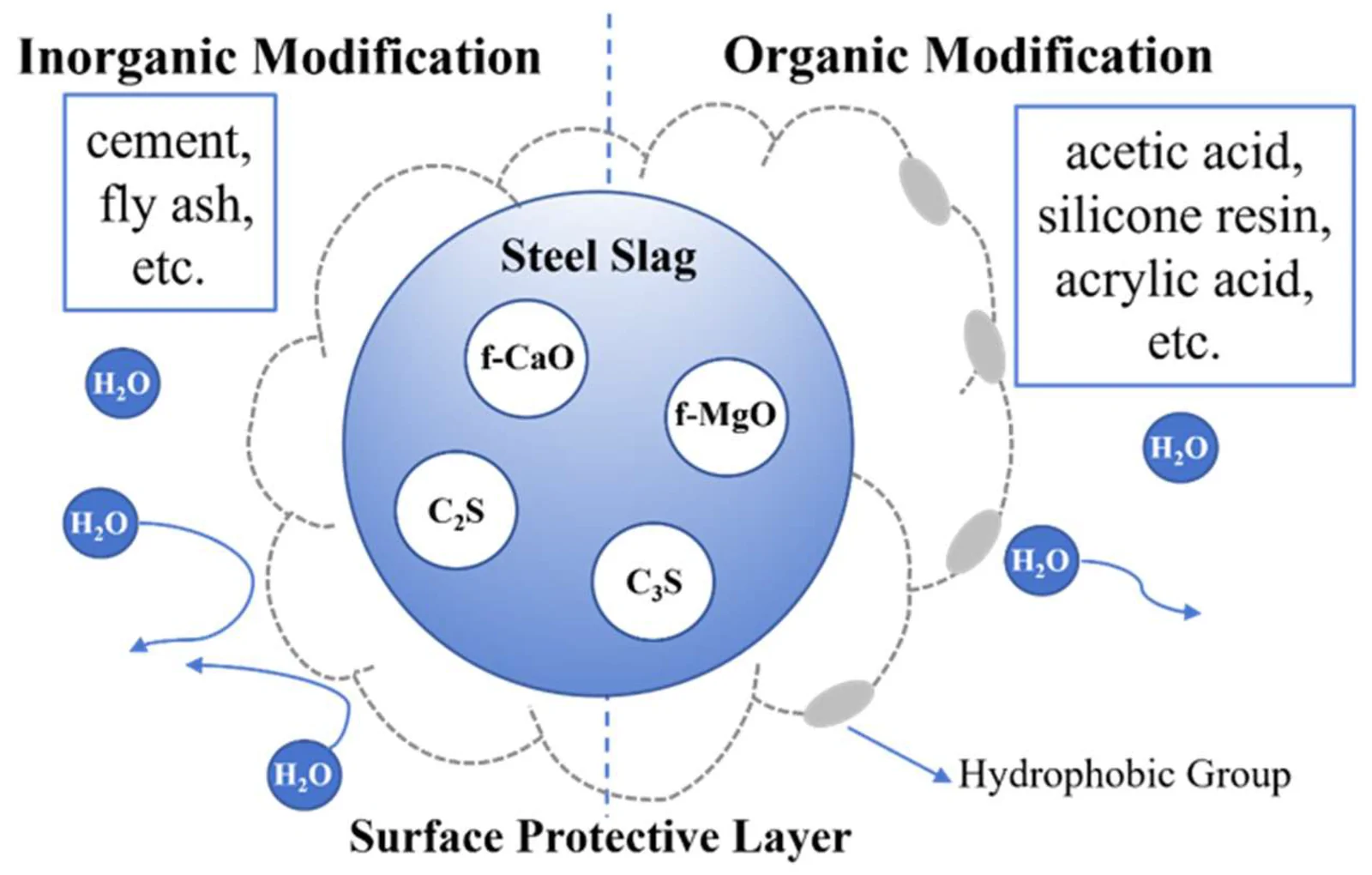
Schematic diagram of the surface modification mechanism.

Surface modification alters the physicochemical properties of the slag surface, including composition, structure, morphology, wettability, and reactivity, without substantially changing the bulk material (Figure 3) [38]. Existing work focuses on chemical methods, which are divided into inorganic and organic approaches [39]. Both modification approaches can regulate the surface structure and composition of SS, suppress the hydration expansion of free calcium oxide, and thereby effectively enhance the volume stability of SS.

Inorganic modification utilizes inorganic materials (e.g., cement and fly ash) to seal surface pores or restrict the diffusion of substances within the material, thus enhancing its performance [40,41,42]. Malathy et al. [40] coated EAF slag with a fly ash-cement slurry. The paste fills and seals surface voids of slag particles to block external moisture ingress and suppress the delayed hydration of internal f-CaO when SS is coated with fly ash-cement slurry. Meanwhile, a pozzolanic reaction takes place between fly ash and calcium hydroxide generated from cement hydration, producing secondary C-S-H gels that reinforce the interfacial bonding between hardened coating matrix and steel substrate. The compact solidified layer formed in this way greatly reduces the chloride ion permeability of slag-incorporated concrete and remarkably elevates its long-term durability. Inorganic coatings have inherent limitations. The bond between the coating and the slag matrix is weak, and coatings can detach during long-term service, re-exposing the f-CaO. The modification process requires drying and curing steps that complicate production and raise costs. Coating also reduces aggregate angularity, weakening the mechanical interlock with the cement matrix and slightly decreasing concrete compressive strength. Critically, this method only provides physical pore sealing and cannot eliminate f-CaO within the slag, so the expansion risk persists if the coating is damaged.

Organic modification employs compounds such as acetic acid and silicone resin to impart hydrophobicity and limit contact between expansive components and water [43,44]. Yao et al. [45] treated SS aggregate with silicone-modified acrylic acid, forming a dense hydrophobic film that sealed surface pores. The treatment reduced water absorption by more than 70% and lowered the volume expansion rate by 35%. Synergistic combinations of modifiers can substantially outperform single agents. Composite systems including hexamethylene diisocyanate biuret with hydroxyl acrylic resin (1.25:1 mass ratio) [46], stearic acid (STA) combined with a silane coupling agent (SCA) [47], and sodium citrate blended with alkali lignin [48] form compact, continuous surface films that seal macropores, micropores, and capillary pores more thoroughly than any single modifier. These multi-component films provide denser coverage, higher hydrophobicity, and stronger interfacial bonding, yielding greater reductions in water absorption and expansion. Nevertheless, two concerns limit organic surface modification. First, protective films may gradually degrade during long-term service, weakening the modification effect and potentially re-exposing reactive phases. Second, the environmental compatibility of surface treatments should be evaluated through appropriate leaching tests, because modification may alter the release behavior of potentially hazardous elements from steel slag [43,49]. Therefore, both the long-term durability of the protective layer and the environmental performance of the treated slag require further evaluation under realistic service conditions.

### 4.2. High-Temperature Modification

Air quenching exploits the low viscosity and surface tension of molten slag at high temperature, enabling fragmentation with low energy input. A high-velocity gas jet is directed at the molten slag stream, dispersing it into fine droplets. The rapid cooling suppresses the formation of coarse f-CaO and f-MgO phases and improves volume stability [50]. During rapid cooling, the morphology, particle size, and reactivity of the free oxide phases are substantially altered. The sharp temperature drop promotes nucleation of f-CaO with reduced particle sizes and a foliated morphology, while MgO nucleation is accelerated, producing finer crystalline phases with particle sizes reduced by roughly an order of magnitude. The altered crystal morphology renders these phases more susceptible to subsequent hydration. The cooling rate also affects the bulk mineral assemblage [51,52,53,54]. Rapid cooling suppresses the β-C2S-to-γ-C2S transformation and favors the retention of metastable β-C2S. It can also limit the decomposition of C3S into C2S and f-CaO by shortening the residence time at elevated temperatures, thereby reducing the formation of f-CaO through this pathway. The RO phase assemblage is also influenced by the cooling rate; however, the direction and magnitude of this effect depend strongly on slag composition and the specific cooling history. However, cooling rate control involves trade-offs. When the MgO content of the slag is high, excessively rapid cooling drives Mg into the RO phase, increasing its MgO content and producing RO phases with poor volume stability [55]. Moreover, quenched slag is extremely hard, so secondary processing, including crushing and grinding, consumes substantial energy and raises the overall cost of utilization. A single cooling method cannot simultaneously optimize all relevant factors. Researchers need to consider both the chemical composition of the slag and the cooling protocol. Recent work has explored using high-pressure, high-velocity CO_2_ gas as the quenching medium, achieving carbonation simultaneously with granulation [56]. This approach enables more efficient stabilization and carbon sequestration in a single step and represents a promising direction.

Hot sealing (hot smothering) is among the established pretreatment routes for steel slag in China [7]. It exploits the waste heat of the slag: water is sprayed into a sealed container, generating large volumes of steam that accelerate the hydration of f-CaO and f-MgO.

**Figure 4:**
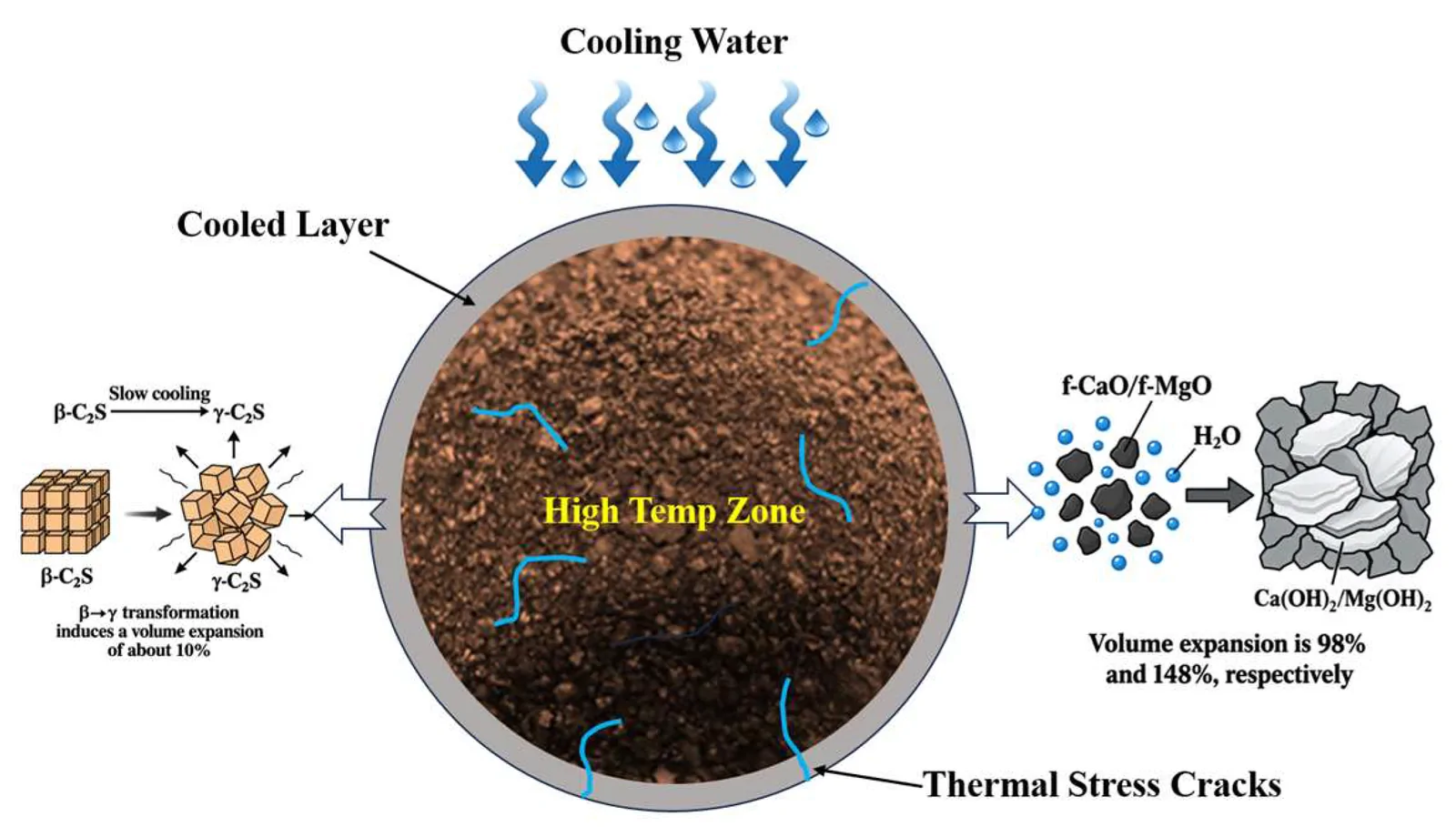
Schematic diagram of physicochemical reactions during the hot sealing process.

The hydration rate of f-CaO depends on steam concentration and temperature. Under the high-temperature, high-humidity, high-pressure conditions inside the sealed vessel, hydration is dramatically accelerated and far more effective than natural aging. Four physicochemical effects operate in parallel (Figure 4): (1) thermal stress induced by sprayed cooling water causes rapid cracking of large slag lumps; (2) saturated steam continuously penetrates into the cracks, while sustained thermal stress promotes further fragmentation; (3) depending on the local cooling history, β-C2S may transform into γ-C2S during slower cooling, accompanied by an approximately 10% volume expansion that contributes to slag disintegration; and (4) rapid hydration of f-CaO and f-MgO under saturated-steam conditions generates additional expansion and cracking.

Hot sealing makes productive use of the slag’s waste heat and effectively mitigates the retarding effect of SS in cementitious systems. The large quantity of water vapor reacts with unstable components during the thermal insulation and stewing period, addressing the volume stability problem before the material enters downstream applications.

### 4.3. Acid Method

**Figure 5:**
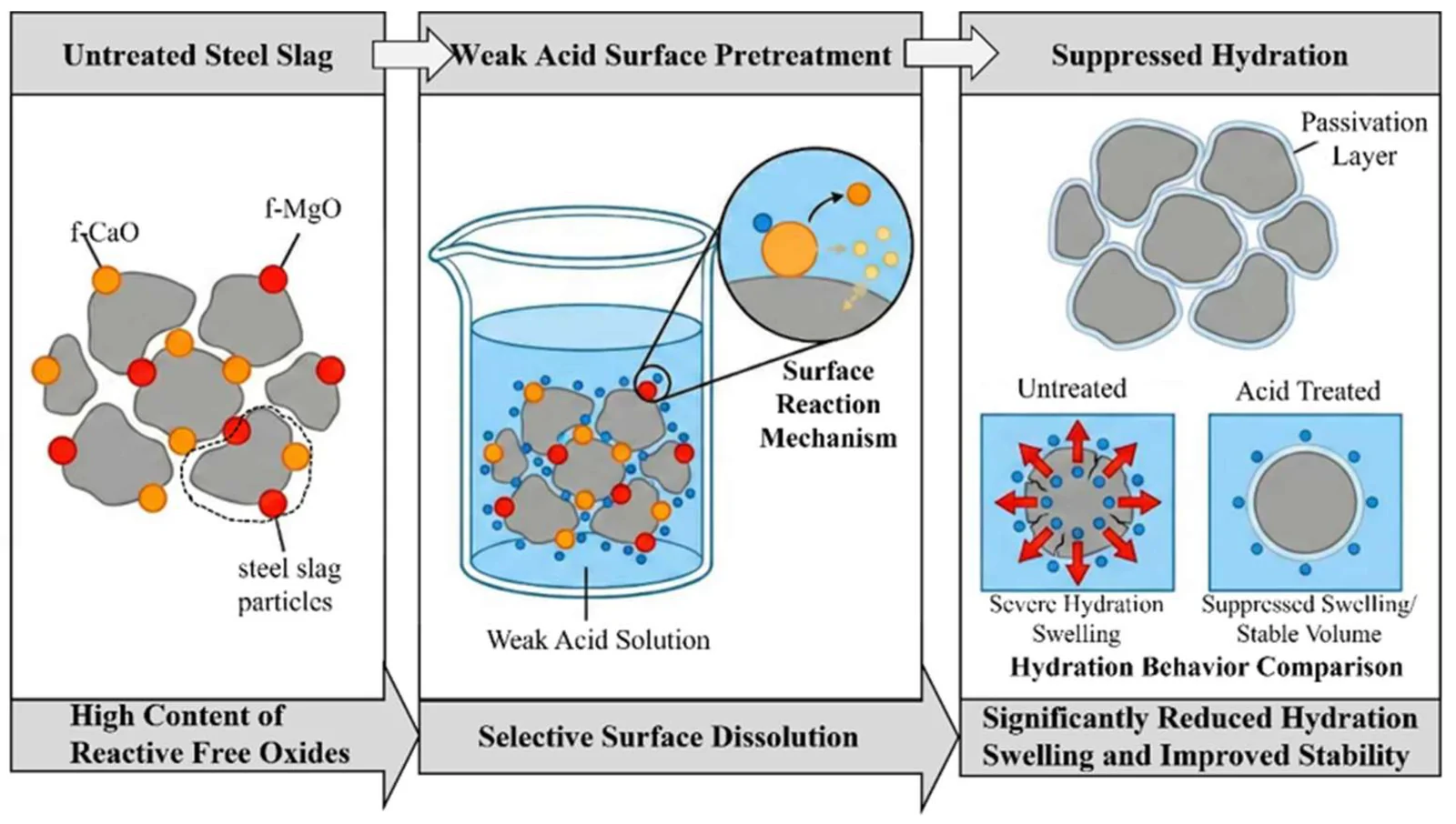
Schematic diagram of the mechanism for SS treatment via the acid method.

Acid treatment converts f-CaO and f-MgO into non-expansive salts through neutralization, substantially suppressing the hydration–expansion pathway of accessible free oxides. As illustrated in the schematic diagram (Figure 5), a weak acid solution triggers selective surface dissolution of exposed free oxides on slag particles, and a dense passivation layer subsequently forms on the slag surface to isolate internal reactive components from external water. The choice of acid involves a trade-off between treatment efficiency and operational practicality. Strong acids such as hydrochloric and sulfuric acid achieve rapid stabilization because of their high proton availability and ability to decompose unstable mineral phases [57], but their corrosivity can damage reaction equipment and create safety hazards. Mild acids including oxalic acid, phosphoric acid, acetic acid, and citric acid have therefore been adopted as practical alternatives that maintain acceptable stabilization performance while reducing equipment corrosion [58].

Acetic acid and oxalic acid react with f-CaO to form calcium acetate and calcium oxalate, which are chemically stable and volume-stable, respectively [59]. Zhao et al. [60] treated SS with acetic acid solutions of varying concentrations and characterized the microscopic composition, morphology, and volume stability of the resulting asphalt mixtures. Compared with untreated SS, acetic acid treatment reduced the content of expansive mineral phases such as f-CaO and promoted the formation of C-S-H, confirming the consumption of unstable expansive components on slag surfaces. Acetic acid pretreatment also optimized the aggregate interfacial structure and improved both volume stability and road performance, with the 15% acetic acid treatment showing the best overall performance. Sun et al. [61] compared acetic and oxalic acid pretreatments for SS asphalt mixtures and found distinct differences in their modification mechanisms and stabilization effects. Acetic acid treatment produced loose flocculent precipitates on the slag surface through neutralization reactions, while the degree of surface erosion increased with increasing acid concentration. In contrast, oxalic acid treatment resulted in a compact layer of blocky calcium oxalate crystals on the slag surface. This layer restricted further contact between the acid solution and internal reactive components, leading to reaction saturation even at relatively low concentrations. Consequently, acetic acid provided continuous improvements in volume stability, high-temperature performance, and water stability with increasing concentration. Oxalic acid reached reaction saturation at a low concentration, with little additional benefit at higher dosages, and its long-term stabilization effect was inferior to that of acetic acid.

Acid treatment protocols must balance modification effectiveness against post-treatment environmental compatibility. The recyclability and harmless disposal of spent acid reagents require careful attention to improve acid utilization efficiency and avoid secondary pollution. Exploring acidic substances derived from other solid wastes for SS modification is a promising direction for achieving “waste-treated-by-waste” and collaborative resource utilization.

### 4.4. Carbonation

**Figure 6:**
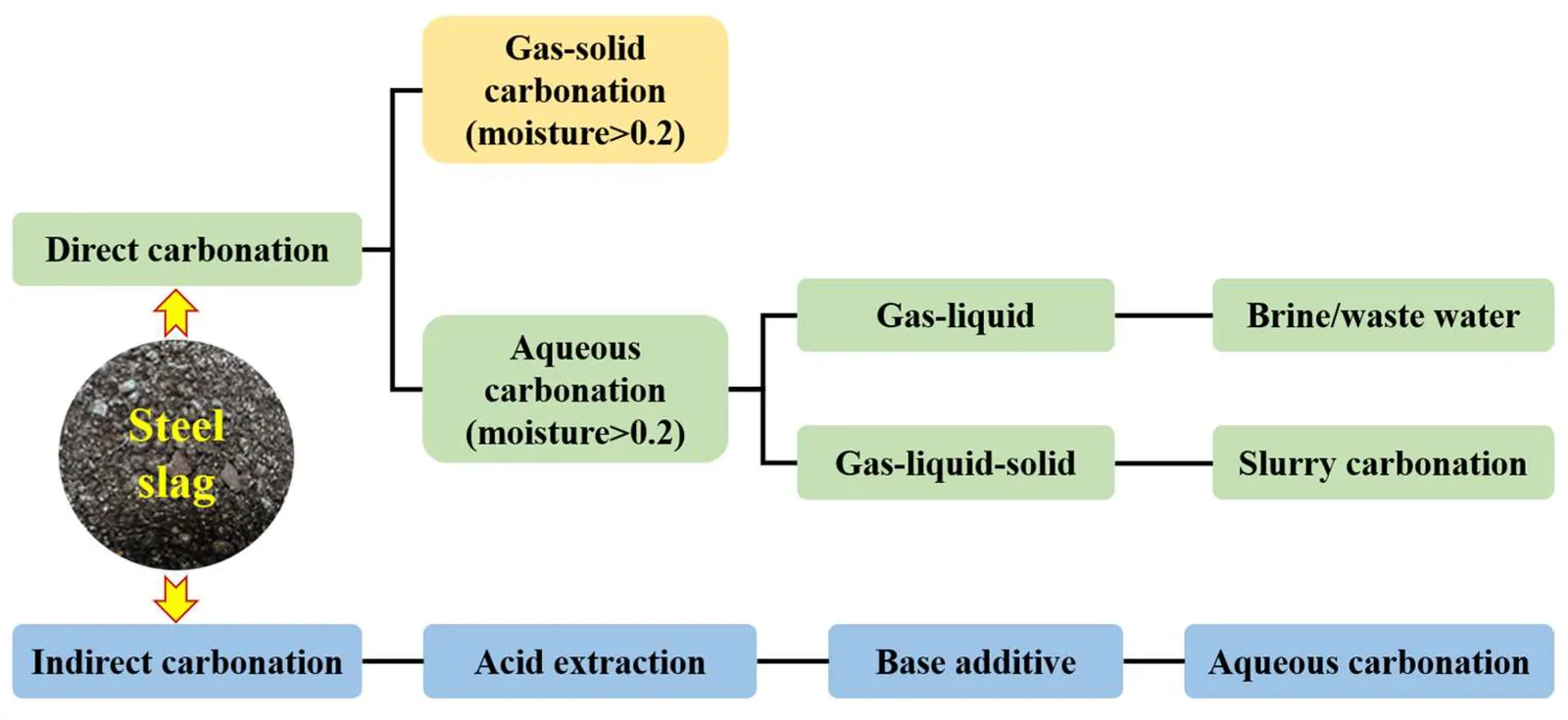
Classification of carbonation technologies for SS.

Carbonation has become an important research direction for steel-slag stabilization because it can simultaneously consume part of the reactive Ca- and Mg-bearing phases and mineralize CO_2_. Nevertheless, the degree of stabilization depends on the extent and depth of carbonation rather than on surface carbonate formation alone [62]. Direct carbonation is further subdivided into dry and wet processes. In dry carbonation, the slag reacts with CO_2_ at elevated temperature under controlled pressure; temperature is the dominant process variable. In wet carbonation, the slag is mixed with a liquid medium to form a slurry into which CO_2_ is introduced; moisture content and humidity are the critical parameters (Figure 6).

The carbonation reactions primarily involve externally supplied CO_2_ reacting with f-CaO, f-MgO, and RO phases in SS to form stable carbonate minerals. Under dry carbonation conditions, free CaO and MgO are converted into stable calcium and magnesium carbonates, thereby reducing the number of expansive phases. Under wet carbonation conditions, reactive minerals such as C_2_S, C_3_S, and C_4_AF can further react with H_2_O and CO_2_ to generate carbonate phases and gel-like products. Mo et al. [63] demonstrated that uncarbonated specimens underwent severe expansion during accelerated curing at 60 °C in water because of the hydration of f-CaO and periclase, whereas carbonated specimens maintained structural integrity and exhibited markedly suppressed expansion. Carbonation products can also form a dense protective layer on aggregate surfaces, providing an additional barrier against subsequent hydration.

The control of CO_2_ pressure, concentration, and reaction temperature is critical to carbonation efficiency [64,65,66]. Excessively high CO_2_ pressure and temperature can accelerate carbonation excessively [67], leading to rapid precipitation of carbonate products on particle surfaces. The resulting dense surface layer restricts further CO_2_ diffusion into the interior of SS particles and consequently limits continued carbonation.

Various additives have therefore been explored as auxiliary agents to optimize the carbonation process, including ethylenediaminetetraacetic acid (EDTA), sodium citrate, sodium tripolyphosphate (STP), and sodium aluminate (SA) [68,69,70,71]. EDTA exerts a concentration-dependent effect on SS carbonation. At moderate dosages, EDTA promotes Ca^2+^ dissolution and carbonate formation, thereby enhancing carbonation and material strength. Excessive EDTA, however, causes over-chelation and rapid carbonate precipitation after CO_2_ introduction. The resulting carbonate layer hinders sustained calcium leaching and ultimately reduces the total yield of calcium carbonate.

Sodium citrate improves carbonation through a different chelation mechanism. It continuously captures dissolved Ca^2+^ to form soluble calcium-citrate complexes, which subsequently react with carbonate ions to precipitate calcite while releasing citrate ions for repeated chelation. In pure water, carbonation tends to produce a relatively dense monohydrocalcite passivation layer that restricts further reaction. In the presence of citrate, fibrous calcite and magnesium-bearing calcite are preferentially formed, producing a more porous surface layer. This loose structure facilitates continued penetration of acidic species into slag particles, sustains Ca^2+^ leaching, and consequently increases the CO_2_ fixation capacity of SS.

Microstructural and elemental analyses further demonstrate the different effects of STP and SA on carbonation [70,71]. In additive-free SS, Ca and Si are mainly concentrated in the particle core, indicating a calcium-silicate-rich matrix, whereas relatively weak Ca and C signals near the particle surface correspond to small amounts of precipitated calcium carbonate. With STP addition, intensive calcium leaching occurs during carbonation, producing a Si-rich outer layer and a substantially greater amount of calcium carbonate around calcium silicate grains. As a result, a thicker carbonate-rich coating is formed on the particle surface. In contrast, SA-modified SS exhibits microstructural and elemental distributions similar to those of the additive-free material. Although partial calcium leaching occurs locally, no distinct Si-rich boundary layer is formed because of the relatively limited carbonation extent. Accordingly, the moderate enhancement in CO_2_ uptake induced by SA produces only minor changes in particle morphology.

EDTA, STP, and SA do not primarily stabilize SS through direct reactions with free oxides. Instead, they improve volume stability mainly by promoting the carbonation of calcium-bearing mineral phases and increasing the formation of stable carbonate products. These products reduce the amount of reactive calcium species and suppress delayed expansion associated with residual f-CaO and f-MgO.

Microbial approaches have also been investigated as alternative carbonation-enhancement strategies [72,73]. Specific microorganisms can promote the transformation of free oxides and silicate phases, thereby improving both the stability and reactivity of SS. Microbial metabolism generates weak organic acids and carbonate ions in situ, which react with f-CaO and f-MgO to form insoluble calcium and magnesium carbonate minerals, reducing the delayed expansion risk associated with residual free oxides. In addition, microbially induced carbonate precipitates can distribute over slag particle surfaces and serve as nucleation sites for subsequent hydration reactions, thereby promoting the reaction of silicate phases and improving the mechanical performance of SS-blended cementitious materials. This strategy provides a promising direction for further development of carbonation treatment.

Despite these advances, a substantial gap remains between laboratory research and practical implementation. Representative studies summarized in recent reviews frequently employ elevated CO_2_ concentrations and well-controlled laboratory conditions [65,66]. For example, Mo et al. [63] employed 99.9% CO_2_ at a pressure of 0.10 MPa for accelerated carbonation. Engineering-scale investigations using industrial flue gas at realistic CO_2_ concentrations and near-ambient pressures remain comparatively limited [65,66].

### 4.5. Comparison of Different Modification Methods

To provide a consistent basis for comparison, the four stabilization routes are evaluated using a common set of criteria, including stabilization depth, reduction in expansive phases or expansion, treatment conditions and duration, mechanical-performance response, energy and reagent requirements, environmental implications, and engineering scalability. Because the available studies involve different slag compositions and testing protocols, these indicators are used to identify technology-specific advantages and limitations rather than to establish a universal ranking.

Surface modification, including inorganic coating and organic hydrophobic treatment, forms a dense coating layer on SS particles and retards the hydration of surface f-CaO and f-MgO, which isolates expansive components from moisture through physical sealing and film formation. It acts rapidly, requires mild conditions, and demands minimal equipment. However, it provides only surface protection without eliminating internal harmful phases. Coatings and films are susceptible to aging and delamination, compromising long-term reliability. Some modifiers reduce the mechanical interlock between aggregate and matrix, lowering concrete strength.

High-temperature modification, including air quenching and hot sealing, utilizes the sensible heat of molten slag to regulate cooling, phase assemblage, and/or free-oxide hydration. Air quenching can refine mineral grains and limit the formation of coarse free-oxide phases, whereas hot sealing accelerates the hydration of accessible f-CaO and f-MgO. However, the effectiveness of these treatments depends strongly on slag composition and process control. Excessively rapid cooling may increase Mg enrichment in RO phases, while quenched slag may require energy-intensive crushing and grinding during downstream processing.

Acid modification can consume accessible f-CaO and f-MgO through dissolution and neutralization, forming more stable Ca- and Mg-bearing reaction products. Weak acids generally offer lower corrosivity and improved operational safety, whereas strong acids provide faster reaction rates but impose greater demands on corrosion-resistant equipment and environmental management. Acid concentration and dosage must therefore be controlled to avoid excessive dissolution of potentially reactive mineral phases, and spent solutions require appropriate recovery or treatment.

Carbonation can consume accessible reactive Ca- and Mg-bearing phases through the formation of stable carbonate products while simultaneously mineralizing CO_2_. Under suitable conditions, carbonation may also promote microstructural densification and improve mechanical performance. Its effectiveness, however, depends strongly on particle size, moisture content, CO_2_ concentration and pressure, temperature, reaction duration, and achievable carbonation depth. Rapid surface carbonation may form a dense carbonate-rich layer that restricts further CO_2_ diffusion and leaves reactive phases in the particle interior incompletely treated.

The suitability of a stabilization route also varies with slag origin. BOFS is generally Ca-rich and may contain relatively high amounts of f-CaO, making treatments that accelerate the hydration or carbonation of accessible Ca-bearing phases particularly relevant. EAFS commonly contains substantial Fe-bearing phases, together with variable amounts of Mg-bearing phases such as RO solid solutions and spinels. Therefore, the stability of Mg-bearing phases and potential environmental leaching require particular attention. LFS generally contains lower Fe and higher Ca–Al contents, with mineral phases such as C_2_S polymorphs, calcium aluminates, and periclase. Depending on its mineralogical composition and cooling history, LFS may undergo self-disintegration associated with the β-C_2_S-to-γ-C_2_S transformation. Accordingly, furnace classification provides an initial indication of treatment suitability, but phase-specific characterization and the determination of free-oxide content should precede the selection of a stabilization method.

Representative quantitative results provide further context for comparing the stabilization performance of these technologies (Table 2). For surface modification, organosilicon-modified acrylic treatment reduced the water absorption of steel-slag aggregate by more than 70.3% and decreased its expansion by more than 35.2% [45]. For high-temperature treatment, increasing the cooling rate from furnace cooling to water quenching reduced the f-CaO content from 14.75 wt.% to 2.64 wt.%, while the calculated autoclaved chalking ratio decreased from 24.98% to 5.84% [51]. For acid pretreatment, acetic-acid-treated steel-slag aggregate exhibited a reduction in the 10 d expansion rate from 1.81% to 0.61%, together with an increase in the freeze–thaw tensile strength ratio from 61.5% to 77.6% [49]. For carbonation, Mo et al. [63] employed 99.9% CO_2_ at 0.10 MPa and reported that 14 d of CO_2_ curing increased the compressive strength of steel-slag concrete by approximately 4.3–5.3 times compared with corresponding specimens after 28 d of conventional moist curing, while also improving volume stability. These values should not be interpreted as directly comparable performance rankings because the slag sources, treatment conditions, specimen types, and testing protocols differ among the individual studies. Comparable quantitative data on treatment cost and industrial-scale applicability are also not consistently reported and are therefore discussed qualitatively.

Environmental performance should be evaluated beyond the direct stabilization effect. Surface treatments involve modifier production and require assessment of long-term environmental compatibility, including the potential release of hazardous elements from treated slag; high-temperature processes can utilize sensible heat from molten slag but may require energy-intensive downstream crushing or grinding; acid treatment consumes reagents and generates spent liquid requiring recovery or neutralization; and carbonation may require CO_2_ capture, conditioning, compression, and gas–solid contacting equipment. Therefore, reported CO_2_ uptake represents gross mineralization rather than net carbon benefit unless the associated material and energy inputs are considered within an appropriate life-cycle boundary.

In summary, no single stabilization technology is universally superior. Carbonation is attractive where an accessible CO_2_ source, adequate gas–solid contact, and sufficient residence time can be achieved, but incomplete penetration may leave reactive phases in the particle interior. High-temperature treatments can be advantageous when molten slag and waste heat are directly available, whereas their effectiveness depends on cooling control and slag composition. Acid treatment can achieve rapid chemical stabilization of accessible free oxides but introduces reagent consumption, corrosion, and spent-liquid management requirements. Surface modification requires relatively mild processing conditions but mainly provides external protection. Technology selection should therefore be based on slag type and mineralogy, required stabilization depth, target application, treatment time, energy and reagent demand, environmental performance, and scale-up feasibility.

**Table 2:** Quantitative comparison of representative steel slag stabilization treatments.

Stabilization Route	Representative Condition	Quantitative Stabilization Indicator	Mechanical/Performance Indicator	CO_2_ Uptake/Environmental Indicator	Main Limitation	Refs.
Surface modification	Silicone-modified acrylic treatment	Water absorption reduced by >70%; volume expansion reduced by ~35%	Interfacial properties improved; effect depends on coating integrity	Environmental compatibility and hazardous-element leaching should be assessed	Mainly surface protection; aging/delamination	[45]
High-temperature modification	Increasing cooling rate to water quenching	f-CaO decreased from 14.75 to 2.64 wt.%; calculated autoclaved chalking ratio decreased from 24.98% to 5.84%	Finer silicate grains; higher retained C_3_S	Uses molten-slag sensible heat, but crushing/grinding demand remains	Cooling control and Mg-rich RO phase	[51]
Acid method	15% acetic-acid pretreatment	10-d expansion reduced from 1.81% to 0.61% in the cited asphalt-aggregate study	Freeze–thaw tensile strength ratio increased from 61.5% to 77.6%	Hazardous-element leaching should be verified; spent acid requires treatment	Reagent use and wastewater generation	[49]
Carbonation	20% CO_2_, 2 bar, 10–120 min; semi-dry or aqueous	Expansion progressively decreased; semi-dry treatment can achieve < 0.5% expansion under reported conditions	30-min semi-dry carbonation increased 3-d compressive strength by ~22.7%	CO_2_ uptake after 120 min: 3.75% (semi-dry), 1.50% (aqueous)	Diffusion/passivation; compression and gas handling	[74]

## 5. The Sustainable Utilization of Modified Steel Slag

SS contains abundant calcium–magnesium minerals and latent cementitious activity, giving it high resource utilization potential. However, poor volume stability and low reactivity have restricted its engineering application. Single modification methods cannot, on their own, substantially raise the overall utilization rate. Research on the sustainable utilization of modified SS is therefore critical for enabling large-scale consumption.

### 5.1. Applications of Modified Steel Slag in Cementitious Materials

Untreated SS exhibits poor volume stability and relatively low cementitious activity in cement-based systems because of the delayed hydration of f-CaO, f-MgO, and other slowly reactive mineral phases. Appropriate stabilization can regulate these reactions, improve volume stability, and, under suitable conditions, enhance the hydration and mechanical performance of SS-containing cementitious materials. Representative treatment conditions and performance indicators reported in selected studies are summarized in Table 3.

Surface modification can simultaneously improve the stability and cementitious performance of SS. Zhang et al. [48] modified SS using a composite system of alkali lignin and sodium citrate and subsequently incorporated the modified SS into cementitious materials. The treatment reduced the water demand, promoted the hydration of SS, and improved mechanical properties. More importantly, the measured expansion was reduced to zero under the investigated conditions, indicating effective suppression of the instability associated with reactive free oxides. However, excessive surface modification may inhibit hydration because of increased electrostatic repulsion between particles.

Acid activation provides another route for improving the soundness and reactivity of SS powder. Zhou et al. [57] used hydrochloric acid pretreatment to modify hot-splashed SS and reported improved surface reactivity, mechanical performance, and autoclave soundness of the resulting SS-blended cementitious system. The improvement was mainly attributed to the removal or transformation of surface f-CaO and the resulting increase in the reactivity of the treated slag. Nevertheless, acid dosage should be carefully controlled because excessive dissolution of reactive mineral phases may adversely affect subsequent cementitious reactions.

Carbonation can improve volume stability while altering the subsequent cementitious behavior of SS. Fang et al. [74] compared semi-dry and aqueous carbonation under controlled CO_2_ conditions and found that the treatment effect depended strongly on carbonation mode and duration. Semi-dry carbonation at an appropriate duration enhanced early-age hydration and compressive strength, whereas excessive carbonation reduced early hydraulic activity. Aqueous carbonation showed a stronger effect on the stabilization of f-CaO and f-MgO and therefore provided greater improvement in volume stability. These results indicate that the carbonation degree should be optimized according to the intended cementitious application rather than simply maximized.

Overall, the representative studies demonstrate that stabilization can improve both the volume stability and engineering performance of SS-containing cementitious materials, but the magnitude of improvement depends strongly on slag characteristics, treatment conditions, and the testing protocol.

### 5.2. Applications of Modified Steel Slag in Aggregates

SS possesses high hardness and a dense texture, giving it considerable potential as a construction aggregate. However, unmodified SS is susceptible to volumetric expansion and structural damage because of its high content of unstable components. After appropriate modification, expansive free oxides can be stabilized and interfacial bonding can be improved, enabling the use of SS as a high-quality aggregate in concrete and road engineering.

In a recent study, SS was modified with stearic acid and a silane coupling agent (KH550), resulting in significant reductions in water absorption and immersion expansion [75]. The treatment effectively suppressed the hydration-induced expansion of f-CaO and f-MgO. The mechanical properties of cement-stabilized modified SS aggregates were also improved. Compared with unmodified SS, the compressive strength increased by 1.6% and 4.4% at 7 d and 28 d, respectively, while the splitting tensile strength increased by 13.9% and 19.2%, respectively. The modified system exhibited a denser microstructure and a substantially lower total pore volume. The interfacial transition zone was also strengthened, as evidenced by a higher proportion of high-density C-S-H gel, more uniformly distributed hydration products, and the absence of obvious interfacial cracks.

Unlike surface coating modification, which primarily forms a protective barrier on slag particles, acid pretreatment can chemically neutralize expansive components within SS and thereby alleviate the volume instability of SS aggregates. During long-term water exposure, continuous hydration of f-CaO and f-MgO in untreated SS aggregates can cause progressive expansion, promote the development of interconnected voids, and deteriorate the internal structure of asphalt mixtures. In contrast, acetic acid treatment neutralizes most of the expansive free oxides within slag particles, thereby suppressing the formation and growth of large interconnected voids and improving structural stability. Among the investigated treatments, 15% acetic acid exhibited the strongest effect, markedly limiting the increase in porosity and average pore volume during prolonged soaking. These results demonstrate that acid modification can effectively suppress the expansion of SS aggregates, reduce the formation of interconnected voids within asphalt mixtures, and enhance the long-term internal structural stability of asphalt pavements containing SS coarse aggregates.

SS coarse aggregates subjected to different pretreatments can also improve the road performance and environmental safety of asphalt mixtures. Sun et al. [49] compared natural aging, hot-water immersion, acetic acid treatment, and silane coupling agent treatment for SS coarse aggregates used in asphalt mixtures. All four treatments significantly improved volume stability. The 10 d expansion rate decreased from 1.81% for untreated SS, which exceeded the specified limit, to 0.94%, 0.86%, 0.61%, and 0.46% after natural aging, hot-water immersion, acetic acid treatment, and silane treatment, respectively. Among these methods, silane treatment exhibited the strongest suppression of hydration-induced expansion associated with f-CaO and f-MgO. Freeze–thaw resistance was also markedly improved, with the tensile strength ratio after five freeze–thaw cycles increasing from 61.5% for untreated SS to 77.6% after acetic acid treatment and 80.1% after silane treatment. All pretreatments maintained the skeletal strength of the mixtures, improved the aggregate-asphalt interface, and reduced the leaching of hazardous elements. Pb leaching was reduced by 32–93%, while As was undetectable in all groups except the natural-aging treatment. All measured values satisfied the relevant environmental requirements.

### 5.3. Engineering Applicability of Different Stabilization Technologies

The engineering applicability of surface modification, high-temperature treatment, acid treatment, and carbonation differs according to slag characteristics, stabilization requirements, processing cost, environmental constraints, and target material performance (Table 4).

Surface modification relies on organic or inorganic coatings to restrict water ingress into slag particles. The process generally operates under mild conditions and requires relatively simple equipment, but the treatment is mainly surface-controlled and its long-term effectiveness depends on coating durability. It is therefore more suitable for applications where shallow stabilization is sufficient, such as selected asphalt mixtures and road-base materials, provided that long-term performance requirements are verified.

High-temperature treatment, including air quenching and hot sealing, can be integrated with molten-slag processing and can utilize the sensible heat generated during steelmaking. These routes are therefore particularly compatible with plants equipped with molten-slag handling and heat-recovery infrastructure and can support relatively large treatment volumes. Their performance, however, depends strongly on slag composition and cooling or steam-control parameters. The potential instability of Mg-rich RO phases and the downstream energy demand for crushing and grinding should also be considered in applications requiring stringent long-term volume stability.

Acid modification can rapidly consume accessible surface free oxides and thereby improve volume stability. Representative studies have also reported improvements in water resistance and freeze–thaw performance under suitable treatment conditions. However, its engineering application is constrained by reagent consumption, equipment corrosion, and the need for recovery or treatment of spent solutions. Consequently, the feasibility of acid treatment should be evaluated on a project-specific basis together with stabilization requirements, cost, and environmental-management capacity.

Carbonation modification can consume accessible expansive Ca- and Mg-bearing phases through the formation of stable carbonate products, while also contributing to microstructural densification and CO_2_ sequestration. Its stabilization efficiency, however, depends strongly on slag mineralogy, particle size, moisture content, CO_2_ concentration and pressure, reaction temperature and duration, and the achievable carbonation depth. Incomplete penetration may leave reactive phases in the particle interior, particularly when a dense carbonate layer forms on the particle surface and restricts further CO_2_ diffusion. Therefore, carbonated steel slag can be considered a promising candidate for low-carbon construction materials, including precast components and other cementitious products, provided that sufficient reaction depth and suitable process conditions are achieved. Its practical suitability should be evaluated together with CO_2_ source availability, energy demand, scale-up feasibility, and long-term engineering performance rather than being regarded as universally superior to other stabilization routes.

**Figure 7:**
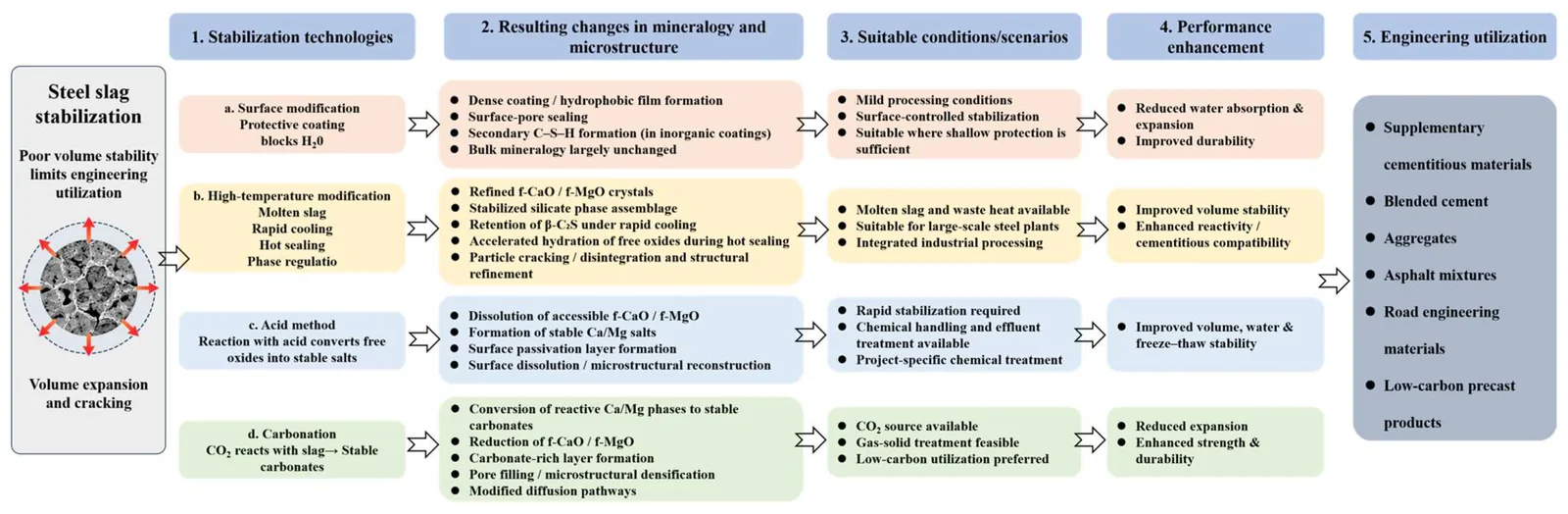
Integrated framework for steel slag stabilization and engineering utilization.

Figure 7 provides an integrated synthesis of Section 3, Section 4 and Section 5 by linking the major stabilization technologies with their resulting mineralogical and microstructural changes, suitable treatment conditions, performance enhancement, and potential engineering utilization. The framework illustrates that different stabilization routes act through distinct mechanisms and are therefore associated with different material responses and application scenarios. Accordingly, the selection of an appropriate stabilization strategy should consider not only the mitigation of volume instability, but also the slag characteristics, treatment requirements, expected performance, and intended engineering use.

## 6. Conclusions and Prospects

### 6.1. Conclusions

This review examined the mechanisms responsible for the volume instability of steel slag and critically compared four major stabilization routes—surface modification, high-temperature treatment, acid treatment, and carbonation—with respect to their mechanisms, stabilization performance, environmental implications, and engineering applicability. The main conclusions are summarized as follows.

(1) Volume instability is governed primarily by the delayed hydration of reactive Ca- and Mg-bearing phases, particularly f-CaO and f-MgO, while the contribution of Mg-rich RO phases and C_2_S polymorphic transformation depends on slag composition and cooling history. The dominant instability mechanism therefore varies among different steel-slag types. BOFS is generally Ca-rich and may contain appreciable f-CaO; EAFS commonly contains higher proportions of Fe- and Mg-bearing RO/spinel phases; and LFS is typically characterized by higher Ca–Al contents, C_2_S polymorphs, calcium aluminates, and periclase. Consequently, stabilization strategies should be selected according to the measured phase assemblage and free-oxide content rather than treating steel slag as a compositionally uniform material.
(2) The four stabilization routes act through different mechanisms and therefore exhibit different advantages and limitations. Surface modification mainly restricts water ingress and delays hydration of reactive phases but does not necessarily consume internal expansive components. High-temperature treatment can regulate mineral phases and accelerate stabilization by controlled cooling or hot sealing, although its effectiveness depends strongly on slag composition and cooling history. Acid treatment can rapidly consume accessible free oxides through dissolution and neutralization, but reagent consumption, equipment corrosion, and spent-liquid management must be considered. Carbonation can convert accessible reactive Ca- and Mg-bearing phases into stable carbonates while simultaneously mineralizing CO_2_, but its effectiveness depends on reaction conditions and achievable carbonation depth. Accordingly, no single stabilization technology can be considered universally superior.
(3) Representative quantitative results demonstrate that all four routes can improve volume stability under appropriate conditions, although direct numerical ranking among studies is not justified because the slag sources, treatment conditions, and testing protocols differ. For example, surface modification has been reported to reduce water absorption by more than 70% and expansion by more than 35%; rapid cooling reduced f-CaO from 14.75 wt.% to 2.64 wt.% in one representative study; acetic-acid pretreatment reduced 10 d expansion from 1.81% to 0.61%; and accelerated carbonation markedly improved both volume stability and mechanical performance under controlled CO_2_ conditions. These results confirm the effectiveness of stabilization treatment but also highlight the need for standardized testing and reporting procedures for meaningful cross-study comparison.
(4) Engineering selection should therefore consider slag type and mineralogy, required stabilization depth, treatment duration, energy and reagent demand, waste generation, environmental performance, available industrial infrastructure, and target application. Surface modification is attractive where mild processing and low equipment demand are prioritized; high-temperature routes are particularly relevant when molten slag and waste heat can be directly utilized; acid treatment may be suitable where rapid chemical stabilization is required and reagent recovery can be managed; and carbonation is a promising option when sufficient gas–solid contact, reaction depth, and an appropriate CO_2_ source are available. However, further engineering-scale validation, particularly under realistic industrial operating conditions, remains necessary before broad generalization of technology performance.

### 6.2. Prospects

Future research directions are proposed below to further promote the green and high-value utilization of SS in construction materials.

(1) Future work should focus on coupled approaches, including hybrid processes combining carbonation and thermal treatment as well as composite modification integrating acid treatment and surface treatment, that combine the strengths of individual methods. The goal is to further reduce residual expansive phases and achieve reliable long-term volume stability under real service conditions, verified by multi-year field exposure rather than accelerated laboratory tests alone.
(2) Low-cost, flue-gas-based carbonation and mineralization induced by microorganisms must be developed to bridge the gap between laboratory studies conducted with pure CO_2_ and the realities of industrial deployment. High-value application scenarios that cover low-carbon concrete, road functional materials, and prefabricated building components should be expanded through pilot-scale demonstrations with rigorous techno-economic analysis.
(3) A unified volume stability evaluation system that incorporates linear expansion, strength retention, visual integrity, and microstructural indicators, together with a long-term expansion prediction model and industry standards, is needed to replace the fragmented, single-indicator methods currently in use. Such a framework would enable consistent comparison across studies, technologies, and slag sources, and would underpin the standardized, large-scale, and sustainable utilization of SS.

## Figures and Tables

**Table 1:** The main chemical compositions of SS (wt.%).

Sample	Types	CaO	Fe_2_O_3_	SiO_2_	Al_2_O_3_	MgO	SO_3_	MnO	Others	Refs.
1	BOFS	43.22	19.99	17.34	4.17	5.91	-	4.60	4.77	[21]
2	BOFS	38.69	18.66	20.15	8.42	6.39	1.09	-	7.69	[22]
3	BOFS	41.00	25.70	13.10	4.20	6.10	0.40	4.20	5.3	[23]
4	BOFS	43.20	12.80	16.80	10.80	8.89	1.15	3.33	3.03	[24]
5	EAFS	28.87	30.49	14.36	9.56	9.90	0.85	1.88	4.09	[25]
6	EAFS	30.89	24.16	17.26	10.71	2.63	0.28	5.68	8.39	[26]
7	EAFS	22.12	36.19	10.11	5.76	8.39	0.37	5.50	11.56	[27]
8	EAFS	26.96	30.99	13.22	13.88	3.78	-	6.41	4.76	[28]
9	LFS	48.10	7.20	20.70	5.60	6.60	1.90	1.30	8.6	[29]
10	LFS	65.68	1.56	8.14	18.03	4.08	1.81	0.17	0.53	[30]
11	LFS	43.65	17.41	22.22	11.16	2.43	0.71	-	2.42	[31]
12	LFS	47.07	9.07	17.10	5.85	5.18	2.18	-	13.55	[32]

**Table 3:** Representative performance of stabilized steel slag in cementitious materials.

Steel Slag	Treatment Conditions	Volume-Stability Performance	Mechanical/Hydration Performance	Refs.
Alkali lignin–sodium citrate modified steel slag	Alkali lignin + sodium citrate modification; 10% SS replacement	Expansion reduced from 1.5 mm to 0	28 d compressive strength reached 49.15 MPa	[48]
HCl-treated steel slag	HCl pretreatment; modified SSP used at 20 wt.%	Autoclave soundness markedly improved compared with untreated SS	7 d and 28 d compressive strength increased by approximately 15%	[57]
Carbonated steel slag	Semi-dry/aqueous carbonation; 20 vol.% CO_2_, 2 bar, 10–120 min; 30 wt.% carbonated SS in mortar	Autoclave expansion reduced; SDC10 reached 0.45%; SDC60 showed no visible cracking	3 d compressive strength of SDC30 mortar: ~39 MPa (approximately 22.7% higher than the reference mortar); 28 d strength changed by less than 5.5%	[74]

**Table 4:** Application scenario comparison of four SS modification technologies.

Modification Methods	Advantages	Limitations	Suitable Factory Scale	Typical Engineering Application
Surface modification	Low investment, simple process, mild conditions	Surface-controlled protection; long-term coating durability requires verification	Small/medium steel plants	Low-dosage asphalt mixture, temporary road base aggregate
High-temperature modification	Utilize molten slag waste heat, on-site synchronous disposal, large slag consumption	Risk of secondary RO expansion for high-Mg slag, strict cooling control	Large long-process converter steel plants	Internal recycling inside steelworks, cement raw material
Acid modification	Rapid stabilization of accessible surface free oxides; improved water and freeze–thaw resistance under reported conditions	High treatment cost, waste liquid disposal burden	All scales (limited to high-budget projects)	Heavy-duty asphalt pavement, hydraulic concrete aggregate
Carbonation modification	Reduction in expansion risk, CO_2_ mineralization, and potential microstructural densification	Slow deep carbonation, carbonate film blocks gas diffusion	Large steel plants with flue gas collection system	Low-carbon precast components, carbon-negative concrete

## Data Availability

No new data were created or analyzed in this study. Data sharing is not applicable to this article.
